# FORTITUDE AND THE MORAL GOOD OF AGING

**DOI:** 10.1093/geroni/igae098.1982

**Published:** 2024-12-31

**Authors:** Nicolai Wohns

**Affiliations:** University of Washington, Seattle, Washington, United States

## Abstract

Fortitude, classically one of the four cardinal virtues, is taken to be necessary for the possession of every other virtue. In this presentation, I analyze the positive aspects of aging from a philosophical perspective and argue that fortitude is also of particular importance for the moral good of aging. Once aging is understood as a dynamic phenomenon with biological, socio-psychological, and existential dimensions, it becomes apparent that it presents myriad physical and mental challenges for which fortitude is required for one to flourish. I focus on the biological dimension in particular, suggesting that, though it is usually defined in terms of loss, biological aging brings with it the possibility for moral virtue. Using this virtue-theoretic framework, I conclude by arguing, contrary to some trans-humanist critics, that there are in fact positive aspects of aging that depend on biological aging.

